# ^18^F-Florbetaben Amyloid PET Imaging: A Chinese Study in Cognitive Normal Controls, Mild Cognitive Impairment, and Alzheimer’s Disease Patients

**DOI:** 10.3389/fnins.2020.00745

**Published:** 2020-07-29

**Authors:** Yan Chang, Can Li, Hui Yang, Yue Wu, Baixuan Xu, Jinming Zhang, Ruimin Wang

**Affiliations:** ^1^Department of Nuclear Medicine, The First Medical Centre, Chinese PLA General Hospital, Beijing, China; ^2^Siemens Healthineers Ltd., Beijing, China

**Keywords:** Alzheimer’s disease, amyloid imaging, florbetaben, PET, amyloid beta

## Abstract

**Objective:**

To evaluate amyloid-β deposition with ^18^F-florbetaben (FBB) PET imaging against ^11^C-PIB PET in cognitive normal controls (NC), mild cognitive impairment (MCI), and Alzheimer’s disease (AD) patients.

**Methods:**

We recruited 45 subjects (15 in each group of NC, MCI, and mild/moderate AD) who had undergone dynamic ^18^F-FBB amyloid PET imaging. For comparison study, 17 participants, including six NC, five MCI, and six AD patients, also underwent ^11^C-PIB PET imaging on separate days. Standardized uptake value ratios (SUVR) were calculated using the cerebellar cortex as the reference region with regions of interest (ROI) manually defined on co-registered CT. Quantitative analysis of mean cortical uptake was calculated using global SUVR. Spearman correlation analysis between MMSE scores and SUVR of 18F-FBB and 11C-PIB images were calculated.

**Results:**

One (7%) of the 15 NC participants, nine (60%) of 15 MCI patients, and 12 (80%) of 15 AD patients had amyloid-positive lesions on ^18^F-FBB PET images. In AD patients, global SUVR was significantly higher than those of MCI patients (1.73 ± 0.62 vs. 1.55 ± 0.11, *P* < 0.001) and NC subjects (1.73 ± 0.62 vs. 1.13 ± 0.43, *P* < 0.001). In the comparison study, one NC participant, five MCI patients, and five AD patients had amyloid-positive lesions on ^11^C-PIB PET images. There was a significant linear correlation (*r*^2^ = 0.81, *P* < 0.001) between ^18^F-FBB and PIB global SUVR values. MMSE scores had negative correlations with SUVR on ^11^C-PIB PET (*r*_1_ = –0.650, *P* = 0.005) or SUVR on ^18^F-FBB PET (*r*_2_ = –0.754, *P* < 0.0001).

**Conclusion:**

Our study suggests that ^18^F-FBB is a useful tracer for the evaluation of amyloid-β deposition in vivo and that global SUVR of ^18^F-FBB PET might be a reliable tool in the diagnosis of AD.

## Introduction

Alzheimer’s disease (AD) is the most common form and the most common cause of dementia in elderly. China has the largest population of patients with AD in the world, accounting for approximately 25% of the entire population with AD worldwide (Jia et al., 2019). Extracellular β-amyloid (Aβ) plaques and intracellular neurofibrillary tangles (NFTs) have been used as AD neuropathologic hallmarks. The regional evolution of AD pathology in terms of Aβ and NFTs has been described in postmortem brain tissue (Braak and Braak, 1991). Aβ load can be quantified using ^11^C-labeled Pittsburgh compound-B (PIB) PET. Neuropathological studies reported that the initial plaques are located in the temporal and orbitofrontal cortices, extending later to the cingulate, frontal, and parietal cortices (Braak and Braak, 1997). In 2011, the National Institute on Aging and Alzheimer’s Association (NIA-AA) created diagnostic guidelines for the preclinical, mild cognitive impairment (MCI), and dementia stages of AD and supported the use of imaging and biomarkers (McKhann et al., 2011). In 2018, NIA-AA updated and unified the new research framework for observational and interventional research of AD. In this research framework, AD is defined by its underlying pathologic processes that can be documented *in vivo* by biomarkers (Jack et al., 2018). Aβ imaging *in vivo* with PET not only allows assessment of Aβ deposition in the brain but also provides an important new tool for the evaluation of the causes, diagnosis, and treatment of dementia (Rowe and Villemagne, 2011).

The most widely used PET Aβ ligand, ^11^C-PIB, was a major breakthrough that provided the first non-invasive *in vivo* detection of cortical deposition and had shown extensive cortical binding in almost all AD patients, indicating that Aβ imaging may help in distinguishing AD patients from healthy elderly controls (Rowe et al., 2007) and differential diagnosis of the dementias (Rabinovici et al., 2007; Rowe et al., 2007). However, the 20-min radioactive half-life of ^11^C limits the use of ^11^C-PIB in research and clinic. To overcome its limitation, a fluorine-18 [18F]-labeled molecule with a radioactive half-life of 110 min is more suitable, which allows widespread distribution from a production facility to multiple sites for research and clinical use. Three ^18^F-labeled Aβ PET tracers have been granted by the US Food and Drug Administration (FDA) and the European Medicines Association (EMA): florbetapir (Amyvid, Eli Lilly), florbetaben (FBB, Neuraceq, Piramal Imaging Ltd.), and flutemetamol (GE Healthcare). The three FDA-approved tracers exhibited different kinetic behaviors and varying levels of specificity with amyloid-β and off-target white matter binding (Landau et al., 2014).

Among the three ^18^F-labeled Aβ PET tracers, ^18^F-FBB was proved to have high in vitro affinity and specificity to amyloid-β, which brought our strong interest. The first human experimental study with FBB for its potential to assess amyloid-β plaques in mild AD patients was carried out in Australia by Rowe et al. (2008). In this study, 15 mild AD patients, 15 healthy elderly controls, and five patients with frontotemporal lobar degeneration (FTLD) underwent ^18^F-FBB PET imaging. Images were analyzed both by visual interpretation (all patients with FTLD and 13 of the 15 HCs as Aβ negative) and by simple semi-quantitative measurement (compared to HCs or FTLD patients, higher neocortical SUVR was observed in AD patients). The results showed a robust separation of patients with AD from healthy elderly controls and FTLD patients (Rowe et al., 2008). In a pivotal histopathology phase 3 study, which validated ^18^F-FBB by comparing *in vivo* PET imaging with postmortem histopathology, the results showed high sensitivity (97.9%) and specificity (88.9%) and high predictive values for the detection of histopathology-confirmed neuritic amyloid-β plaques (Sabri et al., 2015). Results from previous studies (Villemagne et al., 2012; Becker et al., 2013) also support the value of ^18^F-FBB PET as a diagnostic marker.

In China, to the best of our knowledge, such validation of ^18^F-FBB has not been achieved to date, especially for differentiation diagnosis of cognitive normal controls (NC), MCI patients, and AD patients, although ^11^C-PIB and ^18^F-florbetapir were relatively widely used. In our study, the aim was to evaluate and validate brain Aβ deposition in NC and MCI and AD patients using amyloid PET imaging with ^18^F-FBB and in the comparative study using ^11^C-PIB.

## Materials and Methods

### Participants

A total of 45 subjects (15 in each group of NC, MCI, and mild/moderate AD) aged 55–86 years were enrolled in this study. Recruitment and evaluation of NC, MCI, and AD patients were performed at the Department of Neurologic Medicine, Chinese PLA General Hospital. Participants underwent a comprehensive clinical examination including medical history, neurological assessment, routine blood analysis, electrocardiography, psychometric examination, and amyloid PET imaging using ^18^F-FBB and ^11^C-PIB. All participants were given the Mini-Mental State Examination (MMSE) and the Clinical Dementia Rating (CDR) score to evaluate the cognitive status. Of these participants, all NC participants had no impairment in cognition and subjective complaint of memory decline with an MMSE score range of 28 or more and a CDR scale of 0. 15 patients with MCI had objective cognitive impairment and had no disability in their daily lives. The MMSE score was 24–28 and a CDR score of at least 0.5; 15 AD patients met the National Institute of Neurological and Communicative Disorders and Stroke and the Alzheimer’s Disease and Related Disorders Association Alzheimer’s criteria for probable AD and the Diagnostic and Statistical Manual of Mental Disorders-IV criteria for dementia of Alzheimer’s type. Varying from moderate to mild, AD patients were reported with an MMSE score ranging from 18 to 25 and a CDR scale of 0.5 to 2. All participants had completed at least 9 years of education.

All participants underwent structural MRI examinations on a 3T Siemens MRI scanner (MAGNETOM Skyra, Siemens Medical Solutions, Erlangen, Germany). Participants with cerebral infarctions, history of significant head trauma, or brain diseases as well as participants with psychiatric disorders including serious depression and schizophrenia were excluded. Current or recent drug or chronic alcohol dependence or use or any significant other systemic disease or unstable medical conditions were also excluded. The study was approved by the Chinese PLA General Hospital Human Ethics Committee (S2018-166-01). Written informed consent was obtained from all participants or their caregivers before participation. Safety monitoring consisted of clinical symptom observation and intermittent measurement of vital signs. Adverse events and side effects were evaluated 24 h, 48 h, and 2 weeks after injection.

### Tracer Synthesis

^18^F-FBB and ^11^C-PIB were labeled and produced in the Department of Nuclear Medicine, Chinese PLA General Hospital. Xiantong International Pharmaceuticals, Inc. (Beijing, China) supplied the precursor and cold standard for production of ^18^F-FBB. In brief, ^18^F-FBB was synthesized by a PET-MF-2V-IT-I homemade fluoride module. The total synthesis time is about 38 min. The final product had an average specific activity of 337.5 GBq/μmol (222–453 GBq/μmol). The radiochemical purity is over 95%. ^11^C-PIB was synthesized from its corresponding precursors as described elsewhere (Philippe et al., 2011). It was synthesized by ^11^CH3-triflate and 6-OH-BTA-0, then purified by semi-preparative HPLC, and reformulated with a radiochemical purity of >95%.

### PET/CT Imaging

All participants underwent ^18^F-FBB PET/CT in a random order within 6 months after comprehensive clinical examination. PET/CT was performed using a 3D imaging consisting of a PET scanner and a multislice CT (μMl 510, United lmaging, China) in the Department of Nuclear Medicine. A spiral CT for the brain was acquired with CT parameters of 120 kV, 110 mAs, and slice thickness 3.00 mm, equal to those of PET. A vacuum cushion was used to minimize head movement during the scanning.

Participants underwent a dynamic PET emission scan in the three-dimensional mode. Dynamic brain PET images were collected continuously for 20 min. ^11^C-PIB PET/CT images were obtained at 40–70 min after intravenous injection (3.7–5.5 MBq/kg), while ^18^F-FBB PET/CT images were acquired at 90–110 min after injection (3.7–5.5 MBq/kg). Data obtained from the CT scans were used to correct the attenuation for PET emission data.

In a dual-tracer study, ^18^F-FBB and ^11^C-PIB were used in six NC participants and five patients with MCI and six with AD. ^18^F-FBB and ^11^C-PIB PET/CT were performed in all participants on separate days, independently.

### Image Analysis

Regions of interest (ROI) analysis was performed on individual PET images and the co-registered CT images. The standardized uptake value ratio (SUVR) was calculated using the cerebellar cortex as a reference region, due to lack of amyloid plaque. To obtain quantitative regional SUVR values of ^18^F-FBB and ^11^C-PIB PET, circular ROI with a diameter of 1–1.5 cm was placed on eight bilateral cortical regions of the PET images corresponding to anatomic CT scans. The eight bilateral cortical regions were as follows: precuneus cortex (Pre), parietal cortex (PC), anterior cingulate gyrus (ACG), posterior cingulate gyrus (PCG), frontal cortex (FC), temporal cortex (TC), occipital cortex (OC), and cerebellar cortex. Global SUVR was defined as the arithmetic mean of the PC, ACG, PCG, FC, OC, and TC SUVR (Barthel et al., 2011).

For visual analysis of ^18^F-FBB and ^11^C-PIB PET, blind to clinical diagnosis and other clinical data, two independent nuclear medicine physicians with experience in interpretation of ^18^F-FBB and ^11^C-PIB PET images classified the ^18^F-FBB and ^11^C-PIB images as “amyloid positive” or “amyloid negative.” A scan was read as positive if the tracer deposited was visible in one cortical region. A “negative scan” was defined when there was no increased tracer uptake in any cortical region (Marchant et al., 2011). ^18^F-FBB images were generated from the 90–110-min data for visual inspection on a MedEx workstation and displayed with a rainbow color scale. Reading ^18^F-FBB images starting at the cerebellum, scrolling upward to the TC and FC, then to the PCG and Pre, and finally to the PC (Sabri et al., 2015).

### Statistical Analysis

All the data analyses were performed using SPSS (version 25.0; IBM). The normality of the distributions for all continuous variables was tested using the Shapiro–Wilk test. Continuous data for the three groups were evaluated using one-way ANOVA for normally distributed values. For comparisons of SUVR among NC participants, AD and MCI patients were performed using independent-sample *t*-tests. Spearman correlation coefficients and significance levels were used to evaluate correlations between SUVR values of ^18^F-FBB PET vs. ^11^C-PIB PET and MMSE. The effect size was calculated with Cohen *d*. Statistics were considered significant at *p* < 0.05. Data were presented as means ± standard deviations (SDs).

## Results

### Cognitive Function

Fifteen NC, 15 MCI, and 15 AD patients were enrolled in our clinical study between May and October 2019. All the participants completed the study and were included in the final analysis. The participants’ demographic characteristics are summarized in Table 1. There was no significant difference in gender among the NC, MCI, and AD groups. MCI and AD patients were significantly older than NC participants. MMSE and CDR scores were statistically significant among the three groups (Table 1). Remarkably lower MMSE scores (20.80 ± 2.62) and higher CDR scores (1.01 ± 0.42) were observed in AD patients compared with NC participants or MCI patients, while MCI patients showed notably lower mean MMSE scores (25.93 ± 1.28) and higher CDR scores (0.47 ± 0.13) than NC participants.

**TABLE 1:** Demographic information of study participants.

	AD (*n* = 15)	MCI (*n* = 15)	NC (*n* = 15)	*P*-value
Age (years)	74.40 ± 8.33	71.93 ± 7.35	64.07 ± 6.10	–
Gender (M/F)	8/7	7/8	8/7	–
MMSE score	20.80 ± 2.62	25.93 ± 1.28	29.27 ± 0.80	<0.001
CDR score	1.01 ± 0.42	0.47 ± 0.13	0.00 ± 0.00	<0.001

*Data are reported as means ± standard deviation. Abbreviations: AD, Alzheimer’s disease; MCI, mild cognitive impairment; NC, cognitive normal controls; MMSE, Mini-Mental State Examination; CDR, Clinical Dementia Rating Score.*

### Safety Analysis

No serious adverse reaction related to the study drug were observed and reported after the ^18^F-FBB and PIB PET/CT scan and during at least the 2-week follow-up period.

### Visual Analysis

**FIGURE 1:**
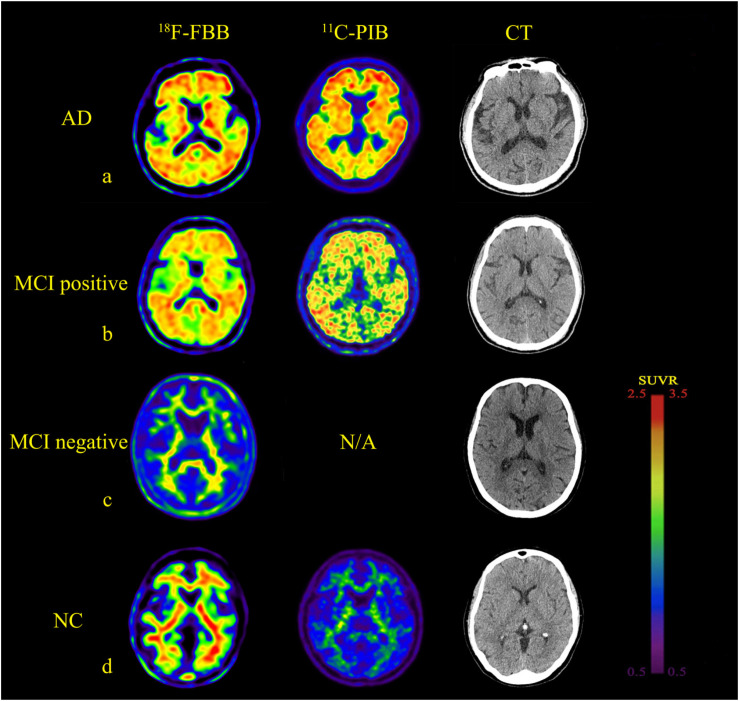
Comparison of brain axial PET images of ^18^F-FBB and ^11^C-PIB in NC, MCI, and AD for example. **(a)** NC participant (75-years-old, male, MMSE = 28). **(b)** MCI patient (73-years-old, male, MMSE = 27). **(c)** MCI patient (67-years-old, female, MMSE = 24). **(d)** Early stage AD patient (78-years-old, male, MMSE = 24). Both ^18^F-FBB PET/CT and ^11^C-PIB PET/CT images showed no amyloid deposition **(a,c)**. Amyloid deposition in widespread cortical was almost identical in ^18^F-FBB PET/CT and ^11^C-PIB PET/CT **(b,d)**.

The ^18^F-FBB uptake was more extensive in Pre, PC, ACG, PCG, FC, TC, and OC (Table 2). A lot of the MCI patients also exhibited extensive Aβ deposition, especially in PCG, FC, and PC. The typical images in AD, positive (and negative) MCI, and NC patients are shown in Figure 1. Compared to the ^11^C-PIB images, higher non-specific binding in the white matter was seen in the ^18^F-FBB images (Figure 1).

**TABLE 2:** Regional and global ^18^F-FBB SUVR and effect size in MCI and AD patients and NC participants.

	^18^F-FBB		
Region	NC	MCI (effect size)	AD (effect size)
Precuneus cortex	1.20 ± 0.28	1.58 ± 0.60(0.92)	1.81 ± 0.43(1.68)
Parietal cortex	1.19 ± 0.20	1.48 ± 0.59(0.65)**	1.75 ± 0.37(1.88)*
Anterior cingulate gyrus	1.08 ± 0.36	1.42 ± 0.69(0.61)**	1.62 ± 0.33(1.56)*
Posterior cingulate gyrus	1.16 ± 0.28	1.63 ± 0.70(0.88)**	1.78 ± 0.41(1.76)*
Frontal cortex	1.01 ± 0.18	1.68 ± 0.93(1.00)**	1.7 ± 0.32(2.65)*
Temporal cortex	1.10 ± 0.26	1.66 ± 0.83(0.91)**	1.76 ± 0.35(2.71)*
Occipital cortex	1.13 ± 0.20	1.48 ± 0.54(0.85)**	1.78 ± 0.34(2.33)*
Global^a^	1.13 ± 0.43	1.55 ± 0.11(1.34)**	1.73 ± 0.62(1.12)*
Cerebellar cortex (reference region)	0.92 ± 0.14	0.97 ± 0.34(0.07)	0.99 ± 0.16(0.19)

Data are presented as means ± standard deviation. Abbreviations: AD, Alzheimer’s disease; MCI, mild cognitive impairment; NC, cognitive normal controls; SUVR, standardized uptake value ratio. *Significantly different from HC (*P* < 0.05). **Significantly different from MCI (*P* < 0.05). ^a^Arithmetic mean of anterior cingulate and posterior cingulate gyrus, parietal cortex, frontal cortex, lateral temporal cortex, and occipital cortex SUVR.

Fourteen of the 15 NC participants were amyloid-negative and clearly distinguishable from patients with AD. One NC participant, a 75-year-old man, with no family history of dementia and no subjective or objective memory decline complaints, with mild positive ^18^F-FBB PET signal, showed increased uptakes in the orbitofrontal cortex and lateral TC as observed in positive ^11^C-PIB PET images. Of the 15 MCI patients, nine (60.0%) had positive scans on ^18^F-FBB PET. Seven of the nine amyloid-positive MCI patients had typical positive scans. The remaining two patients showed focal positive scans: one patient was amyloid-positive in PC, FC, and TC, while the other was amyloid-positive in FC and PC. Among the nine MCI patients, five MCI patients underwent a dual-tracer (^18^F-FBB and ^11^C-PIB) study producing amyloid-positive images in both PET scans. Twelve of the 15 AD patients showed widespread cortical ^18^F-FBB deposition; the proportion of patients with negative scans is consistent with previous clinical samples in AD (Villemagne et al., 2012). Six patients that underwent ^11^C-PIB PET showed an almost identical cortical distribution compared to ^18^F-FBB images. Both ^11^C-PIB and ^18^F-FBB-positive PET images showed highly increased uptakes in CG, FC, TC, PC, and OC, with no appreciable binding in the cerebellar cortex. In three AD patients, a 68-year-old man, a 69-year-old man, and a 72-year-old man, no amyloid plaques deposits were found.

### Quantitative Analysis

**FIGURE 2:**
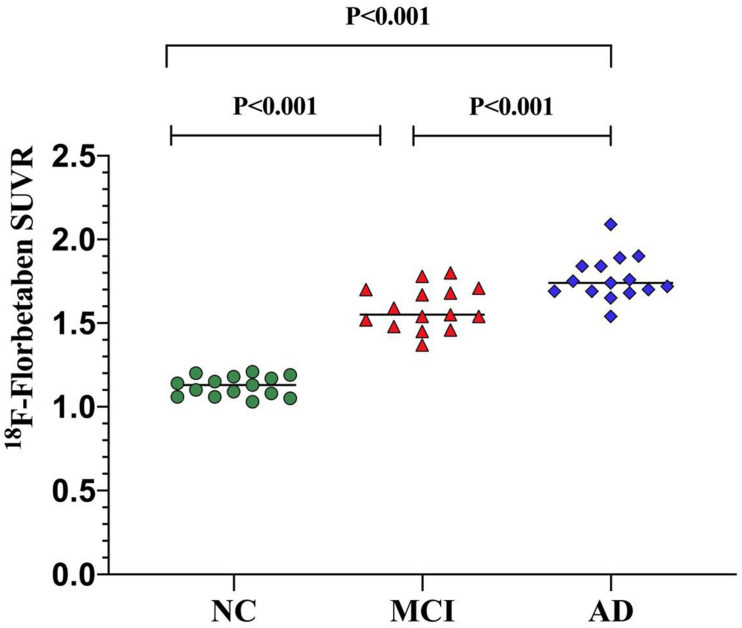
Global SUVR of ^18^F-FBB analyses in NC participants and patients with MCI and AD. Global SUVR of AD patients (1.73 ± 0.62) were significantly higher than those of MCI patients (1.55 ± 0.11) and NC (1.13 ± 0.43) participants.

The mean values of ^18^F-FBB SUVR and effect size by brain regions for the three groups are summarized in Table 2. For ^18^F-FBB PET, the SUVR was significantly higher in AD patients in all cortical regions as well as the global cortex (1.73 ± 0.62; *P* < 0.001) than those of NC participants. There was a significant difference between the global SUVR of MCI patients and NC participants (Figure 2 and Table 2). In ^18^F-FBB PET, MCI patients yielded a slightly higher effect size than did AD patients (*d* = 1.34 and 1.12 for MCI and AD, respectively; Table 2).

**FIGURE 3:**
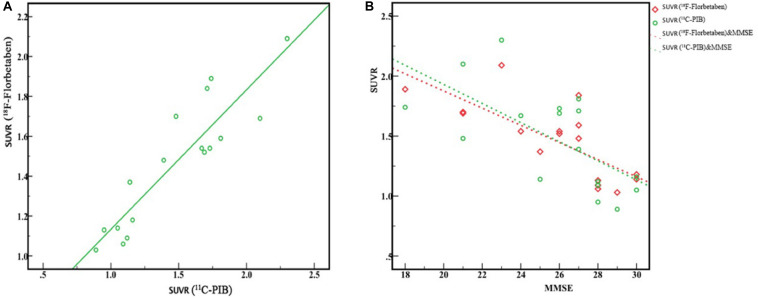
Correlation between ^18^F-FBB and ^11^C-PIB global SUVR from six AD and five MCI patients and six NC participants. **(A)** Excellent linear correlation was observed (*r*^2^ = 0.81, *P* < 0.0001). **(B)** MMSE scores had a negative correlation with SUVR of PIB PET (*r*_1_ = 0.650, *P* < 0.005) and SUVR of ^18^F-FBB PET (*r*_2_ = 0.754, *P* < 0.0001).

Comparisons of characteristics in each group are illustrated in Table 3. Seventeen subjects underwent both ^18^F-FBB and ^11^C-PIB PET/CT studies on separate days, respectively. The final diagnosis was as follows: NC: *n* = 6, MCI: *n* = 5; early AD: *n* = 5; moderate AD: *n* = 1. Global SUVRs in the ^18^F-FBB and ^11^C-PIB PET/CT studies were similar (1.46 ± 0.32 vs. 1.47 ± 0.41, respectively), while ^18^F-FBB SD was slightly wider (Figure 3A). Figure 3A shows between SUVR of ^18^F-FBB PET and global SUVR of PIB PET. A strong positive linear correlation (*r*^2^ = 0.81, *P* < 0.001) of global SUVR between ^18^F-FBB and ^11^C-PIB was observed. Spearman correlation analysis was performed between MMSE scores and SUVR of ^18^F-FBB and ^11^C-PIB images. We found that MMSE scores had negative correlations with SUVR on ^11^C-PIB PET (*r*_1_ = −0.650, *P* = 0.005) or SUVR on ^18^F-FBB PET (*r*_2_ = −0.754, *P* < 0.0001). That is, the higher the MMSE scores were, the smaller the SUVR on ^11^C-PIB PET or ^18^F-FBB PET was. Furthermore, the correlation of the MMSE score with SUVR on ^18^F-FBB PET was detected larger than that with SUVR on ^11^C-PIB PET (| *r*_2_| >| *r*_1_|) (Figure 3B).

**TABLE 3:** Characteristics of 17 participants examined by ^18^F-FBB and by ^11^C-PIB.

Participants	Age	Sex	Clinical diagnosis	MMSE score	CDR score	FBB SUVR	PIB SUVR
1	57	M	NC	29	0	1.03	0.69
2	67	F	NC	29	0	1.18	1.16
3	75	M	NC^a^	28	0	1.13	0.75
4	64	F	NC	28	0	1.06	0.90
5	63	F	NC	30	0	1.14	1.05
6	59	F	NC	30	0	1.09	0.94
7	64	M	MCI	25	0.5	1.37	1.14
8	71	M	MCI	26	0.5	1.52	1.69
9	73	M	MCI^b^	27	0.5	1.59	1.81
10	85	F	MCI	28	0.5	1.54	1.73
11	76	F	MCI	27	0.5	1.48	1.39
12	86	M	Early AD	21	1	1.84	1.71
13	78	M	Early AD^c^	24	1	1.69	2.10
14	76	F	Early AD	23	1	1.54	1.67
15	70	F	Early AD	21	1	1.89	1.74
16	60	F	Early AD	21	0.5	1.70	1.48
17	83	M	Moderate AD	18	2	2.09	2.30
Total	71.0 ± 9.10			25.59 ± 3.66	0.53 ± 0.54	1.46 ± 0.32	1.47 ± 0.41

*Data are reported as means ± standard deviation. Abbreviations: AD, Alzheimer’s disease; MCI, mild cognitive impairment; NC, cognitive normal controls; MMSE, Mini-Mental State Examination; CDR, Clinical Dementia Rating Score; SUVR, standardized uptake value ratio. ^a^Case d, ^b^Case b, and ^c^Case a in Figure 1.*

## Discussion

The aim of this study was to evaluate amyloid positivity by visual analysis and quantitative analysis of ^18^F-FBB SUVR in the NC participants and MCI and AD patients as well as comparing its correlation with ^11^C-PIB PET. As expected, we demonstrated that ^18^F-FBB is a useful amyloid PET tracer with excellent linear correlation in the detectability of amyloid deposition, compared to ^11^C-PIB.

In our study, the AD patients and amyloid-positive MCI patients showed higher cortical uptake than amyloid-negative MCI patients and NC participants in ^18^F-FBB PET/CT. It is important to note in this study that one NC participant was deemed to be amyloid-positive. In addition, the proportion of amyloid-positive patients is lower than in some previous studies (Villemagne et al., 2012; Landau et al., 2014). The prevalence of scans positive of ^18^F-FBB and PIB reported in our study is slightly lower than that of positive scans in NC participants in the previous study (Villemagne et al., 2012). This may be related to the younger age of the participants in the NC group.

In the dual-tracer study, ^18^F-FBB and ^11^C-PIB images were similar to the progression of Aβ deposition categorized by Braak staging (Braak and Braak, 1991, 1997). In the AD spectrum (both MCI and AD), Aβ deposition was seen in FC, ACG, PCG, PC, Pre, and TC in the majority of patients; dual-tracer images may correspond to Braak C stages. There was no significant Aβ deposition in nine patients, which may correspond to Braak stage 0. Visual analysis proved the diagnostic ability of ^18^F-FBB to be comparable to other amyloid PET tracers (Figure 1). The quantification comparison between ^18^F-FBB and ^11^C-PIB exhibited an excellent linear correlation, which further illustrated the diagnostic value of ^18^F-FBB (Figure 3A). Moreover, we found that the threshold of global SUVR was about 1.4 in ^18^F-FBB, which closely corresponded to a global SUVR of 1.5 in ^11^C-PIB. We also found that there was a negative correlation between MMSE score and SUVR values of ^18^F-FBB PET vs. ^11^C-PIB PET in three groups, indicating that the severity of the disease is associated with the significance of cognitive decline.

^18^F-FBB images (Figure 1) showed an excellent differentiation diagnosis possibility in NC, MCI, and AD. The results convince us to believe that this radiotracer provided a robust separation of AD patients from MCI and NC participants. SUVR comparison between three groups verified our hypothesis (Figure 2). This separation was performed by visual image analysis and a quantitative measure derived from a short PET scan. In visual analysis, cortical uptake and global SUVR of ^18^F-FBB were slightly higher in MCI patients than in NC participants in PET scans. The threshold value of global SUVR 1.4 could clearly distinguish between MCI and NC participants. However, several patients with MCI showed amyloid-negative deposition and one patient with amyloid deposition was found in the NC group. It is reported that about 10–30% of healthy aged persons showed high amyloid deposition (Cohen et al., 2012). So, this is reasonable to have an amyloid-positive patient in 15 NC participants. Moreover, for the MCI group, 60% of ^18^F-FBB scans are positive, consistent with the previous study (Anna et al., 2018). Follow-up studies have reported that 70% of amyloid-positive MCI patients will progress to dementia due to AD over 3 years (Okello et al., 2009; Villemagne et al., 2011). Further deep research is needed. As we mentioned above, our clinical diagnosis of AD was not confirmed with pathology due to the unavailability of autopsy.

For visual diagnosis, as shown in Figure 1, a distinct feature was the higher degree of non-specific binding of ^18^F-FBB to white matter, which is a common feature of ^18^F-florbetapir and ^18^F-flutemetamol (Vandenberghe et al., 2010; Wong et al., 2010). Due to the higher non-specific binding in white matter, the visual readouts of ^18^F-FBB and all the other novel ^18^F-labeled amyloid radiotracers seem to be challenging in clinical application. The spillover effect of white matter might result in higher measurement of cortical uptake than its actual value. In NC participants, white matter uptake of ^18^F-FBB was significantly higher than that of ^11^C-PIB. ^11^C-PIB PET images usually show deposition in gray matter in excess of that in white matter in AD. While the ^18^F-labeled amyloid radiotracers all show a distinctive white matter pattern in those with no or low Aβ deposition, in AD patients, these ^18^F-labeled novel tracers frequently show loss of the gray–white matter demarcation with a consequent loss of the normal white matter pattern as the predominant evidence of cortical Aβ deposition (Rowe and Villemagne, 2011). However, each of the FDA-approved agents provides a nearly identical qualitative evaluation of the presence of cortical Aβ deposition and diagnostic abilities (Vandenberghe et al., 2010; Wong et al., 2010; Sabri et al., 2015). Despite this, both radiotracers provided a robust separation of AD patients from MCI-negative patients and NC participants. White matter hyperintensities (WMHs or leukoareosis) are commonly seen in AD patients, and MRI often shows focal hyperintensities in the deep and subcortical white matter. Pietroboni et al. reported that WM damage represents a crucial feature in AD pathogenesis. Moreover, the correlation between CSF Aβ levels and WM-lesion load suggests a confirmed link between Aβ pathology and WM macrostructural and microstructural damage. A further limitation of this study is the lack of structural MRI data, which would allow us to evaluate WM macrostructural and microstructural damage and adjust for volume loss. Besides, SUVR in amyloid PET was calculated using the cerebellum as a reference region in most studies (Barthel et al., 2011; Villemagne et al., 2012; Hatashita et al., 2014) which is known to have very little accumulation of Aβ (Svedberg et al., 2009) leading to a higher SUVR. There was a significant correlation between ^18^F-FBB and ^11^C-PIB in the global SUVR. The slope of the linear correlation was 0.43. This slope is similar to or lower than other ^18^F-labeled Aβ radiopharmaceuticals, such as ^18^F-flutemetamol (slope of 0.81) (Landau et al., 2014), ^18^F-florbetapir (slopes ranging from 0.33 to 0.64) (Wolk et al., 2012; Landau et al., 2013), and ^18^F-FC119S (slope of 0.41) (Byun et al., 2017).

For ^18^F-FBB, PET images are obtained 90 min after injection, similar to ^18^F-flutemetamol (90 min after injection) (Byun et al., 2017) or longer than other ^18^F-labeled Aβ radiopharmaceuticals, such as ^18^F-FC119S (30 min after injection) (Byun et al., 2017) and ^18^F-florbetapir (30–50 min after injection) (Wolk et al., 2012; Landau et al., 2013). Anyway, the total time spent for PET imaging after injection of ^18^F-FBB (110 min) is similar to other ^18^F-labeled Aβ radiopharmaceuticals (40–150 min).

Furthermore, research efforts that have more broadly measured amyloid burden antemortem with amyloid PET imaging might have the potential to be helpful for differential diagnosis in neurodegenerative dementia, including dementia with Lewy bodies (DLB), Parkinson disease with dementia (PDD), and vascular dementia (VaD). Despite these dementias being similar and the overlapping clinical, neuropsychological, and neuropathological features, DLB, VaD, PD, and PDD could be differentiated by their degree of cortical deposition of amyloid and cognitive ability (Rik et al., 2015; Donaghy et al., 2018; Melzer et al., 2019). Some studies found Aβ to be consistently higher among Down’s syndrome (DS); however, the association between Aβ1-40 and Aβ1-42 concentrations among DS and AD dementia was inconsistent (Iulita et al., 2016; Lee et al., 2016; Fortea et al., 2018; O’Bryant et al., 2018). This was the initial study on ^18^F-FBB in China. With little experience for differential diagnosis in neurodegeneration, our future work will explore the role amyloid PET and blood-based biomarkers play in AD and other neurodegeneration dementias during the process of the study and literatures review.

Our study has several certain limitations that should be noted. First, the relatively small number of our study population and the use of correlation instead of regression analysis to interpret data cannot indicate causality between variables, leading to a limited statistical power. Our results should be interpreted with caution. Second, the clinical diagnosis of patients with AD was performed before PET study by certified physicians in a comprehensive diagnosis applying DSM-IV and the NINCDS-ADRDA criteria. No autopsy or histopathological confirmation of amyloid plaque accumulation or biomarkers in cerebrospinal fluids [such as Aβ40, Aβ42, and phosphorylated Tau (pTau)] was performed. Finally, we have not evaluated MCI potential progression to AD. It is possible that several amyloid-positive patients will develop AD in future. Future studies and follow-up study would be required.

## Conclusion

Our clinical study demonstrated that ^18^F-FBB could reliably detect Aβ deposition *in vivo* and discriminating NC and AD patients, which is significantly correlated with ^11^C-PIB. Although ^18^F-FBB showed higher non-specific binding to white matter in participants, we could obtain images similar to ^11^C-PIB PET. The total time spent for ^18^F-FBB imaging is similar to other ^18^F-labeled Aβ radiotracers. In addition, all of these results suggest ^18^F-FBB to be a useful and suitable tool for Aβ deposition imaging *in vivo*. We would like to expand the utilization of this tracer further in various fields of research and clinical practice.

## Data Availability Statement

All datasets presented in this study are included in the article/supplementary material.

## Ethics Statement

The studies involving human participants were reviewed and approved by the Chinese PLA General Hospital Human Ethics Committee. The patients/participants provided their written informed consent to participate in this study. Written informed consent was obtained from the individual(s) and/or minor(s)’ legal guardian/next of kin for the publication of any potentially identifiable images or data included in this article.

## Author Contributions

YC and CL contributed to the conception and design of the study and wrote the first draft of the manuscript. HY performed the statistical analysis. YW revised the manuscript. BX, JZ, and RW ensured that the questions related to the accuracy or integrity of any part of the work are appropriately investigated and resolved. All authors contributed to the manuscript revision and read and approved the submitted version.

## Conflict of Interest

YW was employed by company Siemens Healthineers Ltd., Beijing, China. The remaining authors declare that the research was conducted in the absence of any commercial or financial relationships that could be construed as a potential conflict of interest.
